# Cardioprotective Mechanism of Leonurine against Myocardial Ischemia through a Liver–Cardiac Crosstalk Metabolomics Study

**DOI:** 10.3390/biom12101512

**Published:** 2022-10-19

**Authors:** Weiwei Rong, Jiejia Li, Dingyi Pan, Qinbei Zhou, Yexuan Zhang, Qianxing Lu, Liyun Wang, Andong Wang, Yizhun Zhu, Qing Zhu

**Affiliations:** 1 School of Pharmacy, Nantong University, Nantong 226001, China; 2 Provincial Key Laboratory of Inflammation and Molecular Drug Target, Nantong 226001, China; 3 School of Pharmacy and State Key Laboratory for the Quality Research of Chinese Medicine, Macau University of Science and Technology, Macau 999078, China; 4 Shanghai Key Laboratory of Bioactive Small Molecules, Department of Pharmacology, School of Pharmacy, Fudan University, Shanghai 201203, China

**Keywords:** leonurine, metabolomics, differential metabolites, mechanism, liver–cardiac crosstalk

## Abstract

Leonurine has been shown to have excellent anti-myocardial ischemia effects. Our previous studies suggested that cardiac protection by leonurine during myocardial ischemia appeared to be inextricably linked to its regulation of the liver. At present, however, there are few mechanistic studies of leonurine and its regulation of hepatic metabolism against ischemic injury. In this study, a metabolomics approach was developed to give a global view of the metabolic profiles of the heart and liver during myocardial ischemia. Principal component analysis and orthogonal partial least squares discrimination analysis were applied to filter differential metabolites, and a debiased sparse partial correlation analysis was used to analyze the correlation of the differential metabolites between heart and liver. As a result, a total of thirty-one differential metabolites were identified, six in the myocardial tissue and twenty-five in the hepatic tissue, involving multiple metabolic pathways including glycine, serine and threonine, purine, fatty acid, and amino acid metabolic pathways. Correlation analysis revealed a net of these differential metabolites, suggesting an interaction between hepatic and myocardial metabolism. These results suggest that leonurine may reduce myocardial injury during myocardial ischemia by regulating the metabolism of glycine, serine and threonine, purine, fatty acids, and amino acids in the liver and heart.

## 1. Introduction

Insufficient coronary blood flow causes myocardial ischemia (MI). Prolonged ischemia impairs the delivery of myocardial substrates and oxygen, inducing severe metabolic perturbations in the affected myocardium and ultimately affecting the function of the heart [1]. Thus, at present, metabolic properties of MI have received much attention, and the study of metabolic profiles in the plasma or serum of MI patients has increased [2,3]. However, MI is a systemic process and circulating metabolites may be susceptible to diverse organ systems such as the kidney, brain, and liver. Among these, liver–cardiac interactions during MI have attracted interest due to the critical role of the liver in maintaining systemic homeostasis. For example, the liver is the primary site for lipid metabolism, insulin resistance, and other factors that mediate metabolic syndrome, which are also risk factors for atherosclerotic heart disease [4]. As MI is one of the complications of atherosclerotic heart disease, the effects of liver metabolism during MI should be of particular concern. Thus, studies of hepatic metabolic profiles during MI would be useful in shedding light on the overall mechanism of action of anti-MI drugs at the metabolic level.

Leonurine is an important alkaloid active ingredient in *Leonurus japonicas* Houtt. (also known as *Herba Leonuri* or motherwort), which was recorded in the Chinese pharmacopeia (2020) as an official index to measure the quality of the herb. Leonurine has been reported to be primarily responsible for the anti-MI effect of motherwort. Some studies demonstrated that leonurine showed an anti-apoptotic effect on cardiac muscle cells induced by hypoxia, doxorubicin, or hydrogen peroxide [5,6,7,8] via inhibition of mitochondrial dysfunction [7] and activation of the PI3K/Akt/GSK3β signaling pathway [9]. Leonurine also exhibited excellent antioxidant activity to resist the damage caused by ROS during MI, and decreased the infarct size of the rat heart [10]. Leonurine may partly prevent cardiac fibrosis by regulating the Nox4-ROS signaling pathway [11]. More remarkably, leonurine showed positive effects on some hepatic metabolic diseases closely related to MI. For example, our previous work found that the long-term treatment of leonurine could improve lipid profiles in mice, rabbits, and rhesus monkeys [12]. In phase III trials on patients with hyperlipidemia, leonurine showed a significant hypolipidemic effect via promotion of the reaction converting homocysteine into methionine [13]. The effects of leonurine on these hepatic metabolic processes were previously thought to be part of the mechanism explaining its cardioprotective effects.

The metabolomics technique is an efficient tool to reveal potential etiologies or treatment mechanisms of a drug by providing a global view of the changes in metabolites during different physiological states before and after drug administration. In recent decades, it has been widely used to uncover possible etiological or therapeutic mechanisms. In this study, the metabolomics technique was applied to the cardioprotective mechanism of leonurine on MI. Considering the potential systemic effects of MI and the important role of the liver in maintaining metabolic homeostasis, we investigated not only the effect of leonurine on the cardio-metabolic process, but also its effect on hepatic metabolism during MI. At present, there are few studies on the regulatory mechanism of leonurine on hepatic metabolic disorders during MI. This study could elucidate the cardioprotective mechanism of leonurine.

## 2. Materials and Methods

### 2.1. Materials and Reagents

Leonurine powder with a purity of 98 percent was provided by Zhu Yizhun, a professor from Fudan University. The powder was subsequently dissolved with water to obtain a solution for oral administration.

N, O-bis (trimethylsilyl) trifluoroacetamide (BSTFA) (containing 1% TCMS, *v*/*v*) was purchased from REGIS Technologies Inc (Morton Grove, IL, USA). Methanol was supplied by Merck (KGaA, Darmstadt, Germany). L-2-chlorophenylalanine was purchased from Hengbai Biotechnology Co., Ltd., Shanghai, China. Chloroform, pyridine, and methoxylamine hydrochloride were approved by Beijing Voke Biotechnology Co., Ltd. (Beijing, China). The standard mixture of fatty acid methyl esters (FAMEs) was obtained from Tianjin Alta Technology Co., Ltd. (Tianjin, China), and the distilled water was supplied by Wahaha Co., Ltd., (Hangzhou, China).

### 2.2. Animals and Modeling

The Sprague-Dawley rats (200 ± 20 g) were supplied by the Experimental Animal Center of Nantong University. They were raised ad lib with available food and water in a room with a 12-h light-dark cycle for at least seven days to acclimatize. The room temperature was set at 22 ± 2 °C, and humidity was kept between 45 and 65%.

The procedure of the coronary artery ligation surgery has been described in our previous work [14]. Briefly, the rats were anesthetized and immobilized in a supine position. Electrocardiogram (ECG) monitoring was performed during the surgery. Endotracheal intubation and artificial ventilation were then performed. The intercostal muscles of the rat were separated and a thoracotomy was performed at the third intercostal position along the left margin of the sternum at 0.5 cm. The pericardium was cut away to expose the heart. The left anterior coronary artery was permanently pinched by a filiform needle between the left atrial appendage and pulmonary artery cone, at approximately 2.3 mm from the root of the aorta. The incision was sutured to allow the rat to breathe spontaneously. The rats with coronary artery ligation surgery were selected and randomly divided into two groups: the MI (M) group (*n* = 7) and leonurine administration (L) group (*n* = 7). The rats in the sham operation (S) group (*n* = 7) performed the same operation except for the coronary ligation. Forty-eight hours after the coronary ligation, the rats in the L group received orally administrations of leonurine (60 mg/kg/d) solution for eight weeks, and the remaining two groups were given the same dose of distilled water. At the end of the eighth week, the myocardium and liver tissues of the rats were collected and stored at −80 °C. The animal study was approved by the Institutional Animal Care and Use Committee, Nantong University. Animals were maintained in accordance with the regulations of the Experimental Animal Administration, issued by the State Committee of Science and Technology of the People’s Republic of China.

### 2.3. Sample Preparation

About 0.1 g of myocardial or hepatic tissue was added to 0.4 mL of a methanol–chloroform (3:1) mixed solution and 20 μL L-2-chlorophenylalanine solution (1 mg/mL stock in dH_2_O) as the internal standard. The mixture was then vortexed for 30 s, followed by homogenization in a spherical mill at 65 Hz for 3 min. The mixture was centrifuged at 10,000× *g* for 15 min at 4 °C. A supernatant of about 0.35 mL was transferred to a vacuum concentrator and dried at 37 °C. Quality control (QC) samples were prepared by mixing equal aliquots of supernatant from each sample. The residues were dissolved in a volume of 80 μL methoxylamine hydrochloride (dissolved in pyridine, 20 mg/mL), and then incubated at 80 °C for 20 min after mixing and sealing. The lid was opened and 100 μL BSTFA was added into each sample, then they were sealed again and incubated at 70 °C for an hour. The mixture was cooled to room temperature, and then a volume of 10 μL FAMEs (C8-C16: 1 mg/mL; C18-C24: 0.5 mg/mL in chloroform) was added. The mixture was well-mixed for GC-MS analysis.

### 2.4. Instruments, Parameters, and Conditions

Gas chromatography/time-of-flight mass spectrometer (GC/TOF-MS) analysis was performed using an Agilent 7890 GC system, coupled with a Pegasus HT TOF-MS. The separation was achieved by an Rxi-5Sil MS column (30 m × 250 μm, 0.25 μm). A 1 μL aliquot of the analyte was injected into the system. Helium was used as a carrier gas. The pre-injection purge flow rate was set to 3 mL/min and gas flow rate through the column was set to 1 mL/min. The initial temperature was set as 50 °C, and kept for 1 min, then raised to 330 °C at a rate of 10 °C/min, then kept for 5 min at 330 °C. The temperature of both the injection and transfer line was set at 280 °C, and the ion source temperature was set at 250 °C. The energy was −70 eV in the electron impact mode. Mass spectrometry data were acquired in full-scan mode with m/z ranging from 30 to 600 Da at a rate of 20 spectra per second, after a solvent delay of 366 s.

### 2.5. Statistical Analysis

The raw data was processed by Chroma TOF 4.3X software and LECO-Fiehn Rtx5 database (c), including raw peak extracting, data baseline filtering, baseline calibration, peak alignment, deconvolution analysis, peak identification, and peak area integration. The missing values were replaced by half of the minimum values. After Pareto scaling and log transformation, the performance of different groups was evaluated by principal component analysis (PCA) and orthogonal partial least squares discrimination analysis (OPLS-DA) in SIMCA software (version 14.1; umetrics). The metabolites with variable importance on projection (VIP) values of more than 1 were considered as differential metabolites [15]. Hierarchical cluster analysis (HCA) was performed by MetaboAnalyst 5.0 software, and the metabolic signal pathway was analyzed by KEGG database (http://www.genome.jp/kegg/, accessed on 26 August 2022). Statistical analysis was performed using a Student’s *t*-test and metabolites with *p*-values less than 0.05 were classified as having statistically significant differences. Correlations of differential metabolites in both myocardial and hepatic tissues were analyzed using debiased sparse partial correlations, and the results were visualized using Metscape 3.1.3 software. The retention time index (RI) method was used in peak identification, and the RI tolerance was 5000.

## 3. Results

The results of pathology showed that leonurine significantly reduced the infarct size in rats induced by MI. In addition, we observed significant decreases in creatine kinase-muscle/brain (CK-MB) activity and troponin I (Tn-I) concentration in the plasma of rats treated with leonurine, and these two indicators are relevant to MI [16,17]. Our results suggest a clear protective effect of leonurine on MI-induced myocardial injury. Details are provided in Figure S1 in the Supplementary Materials.

**Figure 1:**
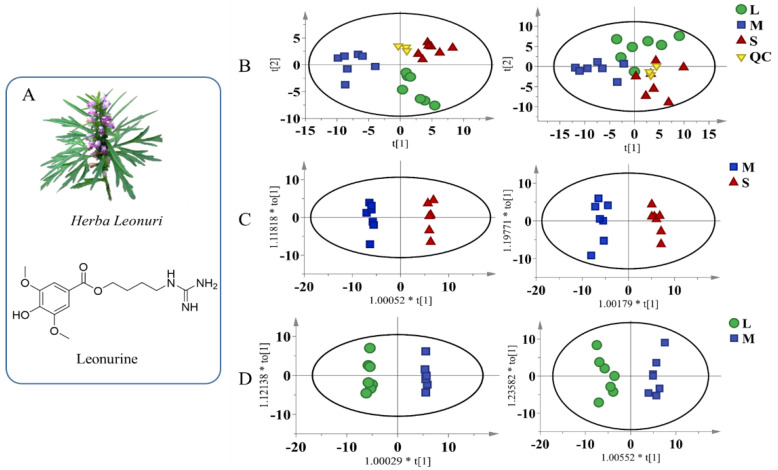
(**A**) The structure of leonurine; score plots of metabolomics for myocardial or hepatic tissue samples. (**B**) The PCA score plots of QC, S, M, and L groups in myocardial (R^2^ = 0.444) and hepatic tissue (R^2^ = 0.478). (**C**) The OPLS−DA score plots of the myocardial (R^2^ = 0.996, Q^2^ = 0.910) and hepatic tissue (R^2^ = 0.975, Q^2^ = 0.844) from S and M groups. (**D**) The OPLS−DA score plots of the myocardial (R^2^ = 0.998, Q^2^ = 0.851) and hepatic tissue (R^2^ = 0.948, Q^2^ = 0.710) from M and L groups, *n* = 7. The yellow inverted triangle, red triangle, blue diamond and green circle represent the QC, S, M, and L groups, respectively. QC: quality control samples; S: sham−operated group; M: myocardial ischemia model group; L: leonurine administration group.

The QC samples of myocardium and hepatic tissues were distributed closely to the center of the PCA score plots (Figure 1A). The samples of the M group were significantly separated from the S group, indicating that serious metabolic disorders occurred in myocardial and hepatic tissues during MI. Compared with the M group, the distribution of L group samples approached the samples in the S group, suggesting that the levels of some metabolites recovered after oral administration of leonurine. The R^2^ value of PCA was 0.444 for myocardial tissue and 0.478 for hepatic tissue, indicating that the cumulative explanatory rate of the model is good.

**Figure 2:**
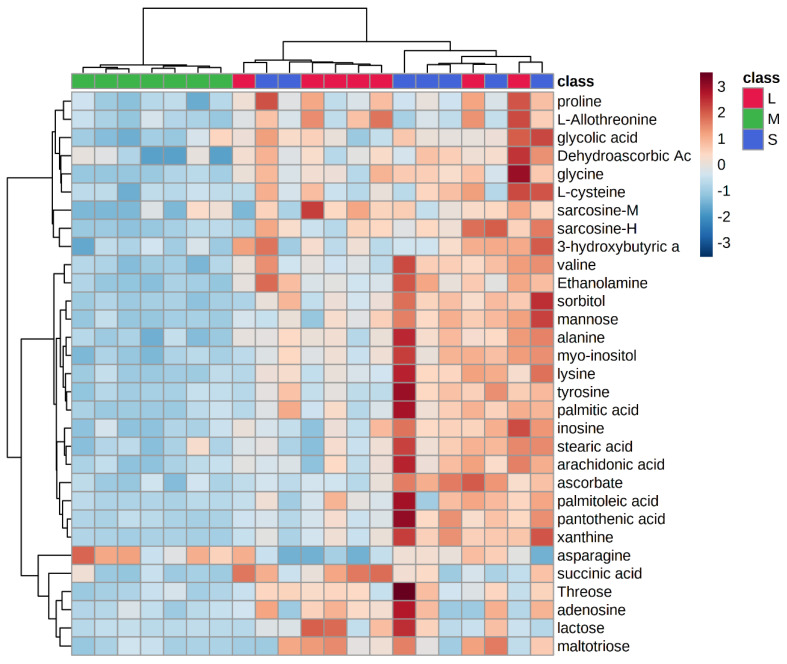
Hierarchical clustering heatmap showing changes in differential metabolite content in the plasma of rats in the S, M, and L groups. The three groups achieved basic separation based on thirty-one differential metabolites. Sarcosine−M indicates sarcosine detected in the myocardium tissue, and sarcosine−H indicates sarcosine detected in the hepatic tissue.

**Figure 3:**
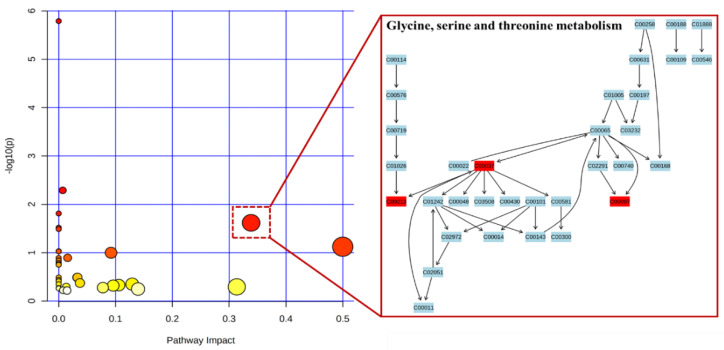
An overview of pathway analysis of thirty-one differential metabolites. The colors varying from yellow to red means the metabolites have different levels of significance. The size of the circles represents the impact value of the pathways. Glycine, serine, and threonine metabolism with an impact value of 0.3387 and *p* value of 0.02414 was considered important. C00213: sarcosine; C00037: glycine; C00097: cysteine.

Some OPLS-DA models were also established to filter the differential metabolites between groups. In the score plots of OPLS-DA in Figure 1B,C, complete separation was achieved between the S and M groups, as well as between the L and M groups. The values of R^2^ were greater than 0.948, and Q^2^ were greater than 0.710 in both myocardial and hepatic tissues. The results of the permutation test for the OPLS-DA model were shown in Figure S2. As a result, six differential metabolites were detected in myocardial tissue and twenty-five in hepatic tissue; details are listed in Table S1. In the HCA results (Figure 2), the samples of the M group were separated from the samples of the S group, indicating disorders of metabolism during MI. The related pathways analysis of thirty-one differential metabolites were shown in Figure 3. All matched pathways were filtered based on *p* values from the pathway enrichment analysis and pathway impact values from the pathway topology analysis. In particular, glycine, serine, and threonine metabolism with an impact value greater than 0.1 and a *p*-value less than 0.05 should be given additional attention.

**Figure 4:**
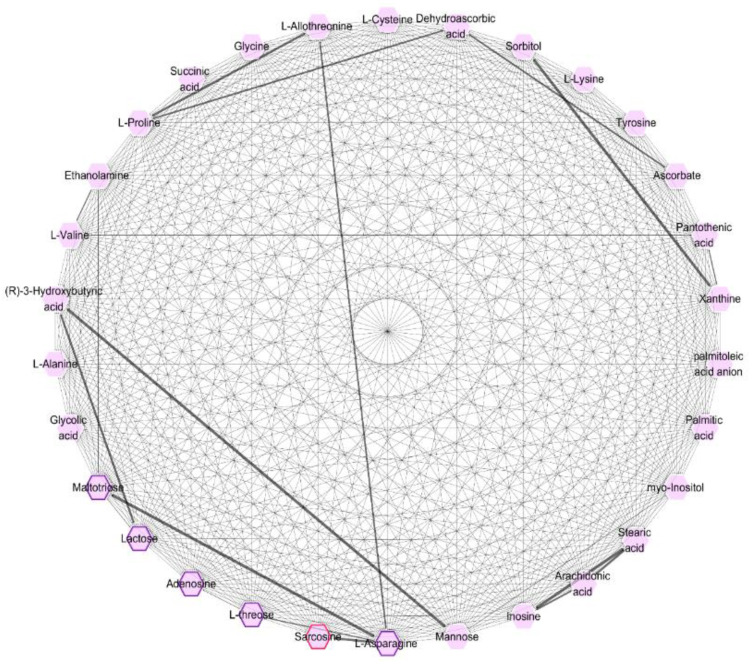
Correlation analysis between six differential metabolites in myocardial tissue and twenty-five in hepatic tissue. Six differential metabolites in the myocardial tissue were framed by a purple line. Sarcosine was detected in both myocardial and hepatic tissue, which was framed by a red line.

The correlation analysis is shown in Figure 4 for thirty-one differential metabolites. Among the differential metabolites, sarcosine was observed in both myocardium and liver tissue. Maltotriose and lactose in myocardial tissue were shown to be significantly correlated with ethanolamine and 3-hydroxybutyric acid in hepatic tissue, respectively. In addition to the obvious correlations with other myocardial metabolites (sarcosine, threose, and maltotriose), asparagine in myocardium tissues also showed correlation with allothreonine, which was a differential metabolite detected in hepatic tissues. These results indicated a crosstalk between the heart and liver during prolonged MI.

## 4. Discussion

In this study, we found more differential metabolites in the liver tissue of rats with long-term MI than in the myocardial tissue, suggesting a significant role of liver metabolism in long-term MI. Of the 31 potential differential metabolites, sarcosine attracted particular attention, due to the disorder observed in both myocardial and liver tissue. As a glycine transporter type 1 (GlyT-1) inhibitor, sarcosine can induce ischemic tolerance against global cerebral ischemia via changing glycine transport [18]. Glycine is a well-documented cytoprotective agent, which offers protection against ischemia-reperfusion injury in vivo in multiple organs, including myocardial tissue [19]. In addition to sarcosine, disorders in glycine and cysteine were also observed in the liver tissue of rats with MI in this work. These three metabolites were involved in glycine, serine and threonine metabolism, which is considered the most significant pathway, according to the results of our pathway analysis (Figure 3). Sarcosine was also reported to reduce the expression of the N-methyl-D-aspartate receptor (NMDAR) in the brain [18]. In addition to the brain, NMDAR is also expressed in cardiomyocytes, and NMDAR-driven calcium influx promotes apoptosis in ischemic human cardiomyocytes [20]. Levels of sarcosine, glycine, and cysteine increased after oral administration of leonurine, suggesting that leonurine inhibits MI-induced apoptosis in cardiomyocytes by modulating the glycine, serine, and threonine metabolism pathways.

As shown in Figure 4, sarcosine has a close correlation with asparagine, and an obvious increase in asparagine was observed in the myocardium tissue of rats with MI. The phenomenon was consistent with the result of a previous metabolomics study on sudden cardiac death [21]. The change in asparagine levels may be the net result of changes in metabolism, transport, and rate of protein and nucleotide synthesis and degradation during MI [22]. Unlike asparagine, other myocardial metabolites such as threose, maltotriose, lactose, and adenosine decreased in the myocardium tissue of rats with MI. Among them, adenosine is the core structure of the energy transfer molecules, adenosine diphosphate (ADP) and adenosine triphosphate (ATP). In addition to energy transfer, adenosine is also a potent vasodilator, which can improve myocardial salvage, reducing infarct size in MI patients and preserving endothelial function [23,24]. Thus, it is likely that the regulation of adenosine by leonurine contributes to the reduction in ischemic damage to the myocardium.

In addition to adenosine, disorders of other metabolites in purine metabolism, such as xanthine and inosine, have been observed in liver tissue. Xanthine, a downstream product of adenosine in purine metabolism, is another indication of the heart–liver interaction during MI. Inosine dilates coronary blood vessels and increases blood flow, resulting in reduced myocardial damage. It also exhibits protection against ATP loss during ischemia and improves functional recovery in reperfusion. In addition, inosine may influence carbohydrate uptake, the activity of glycolytic enzymes, and release of insulin [23]. Summarizing the above, it is likely that the effect of leonurine on the purine metabolism pathway plays an important role in its myocardial protection during ischemia.

Disorders in the metabolism of fatty acids, including palmitoleic, palmitic, stearic, and arachidonic acids, have also been observed in liver tissue. Among them, stearic and arachidonic acids show a significant correlation with inosine in Figure 4, suggesting a relationship between fatty acids and purine metabolism. Stearoyl-CoA desaturase 1 (SCD-1) regulates the desaturation of saturated fatty acids (mainly stearic and palmitic acids) to monounsaturated fatty acids [25], and palmitoleic acid is one of the main products of the desaturation by SCD-1. In humans and rodents, SCD-1 is mainly expressed in liver and adipose tissue, and its expression can be modulated by sulfur amino acids (e.g., cysteine) [26]. In our previous study, the regulation of leonurine on other sulfur amino acids (homocysteine and methionine) was reported [13]. Methionine, homocysteine, and cysteine metabolism are highly correlated and have been reported to play a role in the regulation of lipid metabolism. Thus, the regulation of leonurine on the cysteine level in this work probably affected the activity of SCD-1, which further influenced the level of the fatty acids (stearic, palmitic, and palmitoleic acids). The change in arachidonic acid levels was related to the expression of sterol regulatory element-binding protein (SREBF) [27]. Interestingly, in our previous study of leonurine on the treatment of atherosclerosis, we found that leonurine suppressed gene expression of fatty acid synthesis, such as SCD-1 and SREBF in liver tissues [12]. From this, we speculate that the regulation of fatty acids by leonurine in this study may be achieved by modulating the activity of SCD-1 and SREBF. In addition, the arachidonic acid pathway is closely related to pathophysiological processes such as inflammation, oxidative stress, and apoptosis during ischemia [28]. The regulation of the arachidonic acid pathway by leonurine is presumably one of the effective ways in which it exerts its antioxidant and anti-inflammatory effects [29]. Changes in hepatic fatty acids levels can also influence the level of acetoacetic acid (a downstream oxidation product of fatty acids), which, in turn, is converted to 3-hydroxybutyric acid. Thus, presumably influenced by changes in fatty acids, a significant downward trend in 3-hydroxybutyric acid was also observed in the rats of group M in this study. According to the study of Nakatani et al. [30], 3-hydroxybutyric acid acts as a kind of ketone body, probably involved in myocardial energy provision. Figure 4 also shows a correlation between 3-hydroxybutyric acid and lactose in myocardial tissue, which may also be another indication of the involvement of 3-hydroxybutyric acid in the myocardial energy supply.

Amino acids are important alternative substrates for ischemic myocardium during energy crises, due to their potential for non-oxidative metabolism and lower contribution to acidosis. In this study, abnormal metabolism of various amino acids (valine, alanine, glycine, proline, tyrosine, lysine, and allothreonine) was also observed in liver tissue. Among them, valine has been demonstrated to reverse electrophysiologic changes caused by metabolic inhibition and hypoxia. Biochemically, during energy crisis/ATP depletion, the degradation process required to convert valine into succinyl coenzyme-A via propionyl and methylmalonyl coenzyme-A is initiated by transamination, followed by a rate-limiting decarboxylation dehydrogenase reaction. The succinyl coenzyme A then undergoes deacylation to yield succinic acid and GTP. In this way, valine offers cardioprotection during hypoxia resulting from MI [31]. Proline and its metabolism impact cell survival and death outcomes by influencing the cellular redox state and maintaining cellular energy under oxidative and nutrient stress conditions, contributing to the tricarboxylic acid cycle (TAC) and glutathione (GSH) biosynthesis. The glutamic acid produced by the oxidation of proline enters the TAC after being converted into α-ketoglutarate [32]. The conversion of alanine to pyruvate and α-ketoglutarate to glutamic acid via alanine aminotransferase is a major pathway to fulfill the energy needs of cardiac myocytes [22,31,33]. In summary, leonurine may affect energy metabolism by regulating the metabolism of amino acids.

Leonurine also alleviates significant reductions in sorbitol during ischemia. The polyol pathway converts glucose to sorbitol in a reaction catalyzed by aldose reductase, with nicotinamide adenine dinucleotide phosphate (NADPH) as the cofactor. Sorbitol is then oxidized to fructose by sorbitol dehydrogenase (SDH) using NAD^+^ as a cofactor. The impact of flux catalyzed by SDH can limit the amount of NAD^+^ available for glyceraldehyde-phosphate dehydrogenase (GAPDH), thus limiting glycolysis and glucose use. As an ischemic heart is heavily dependent on glycolysis to generate ATP, flux via SDH would affect ATP levels during ischemia, and hence, cause injury to the myocardium [34]. Thus, decreased regulation of sorbitol by leonurine indicates its positive effect on glycolysis and glucose use during MI.

**Figure 5:**
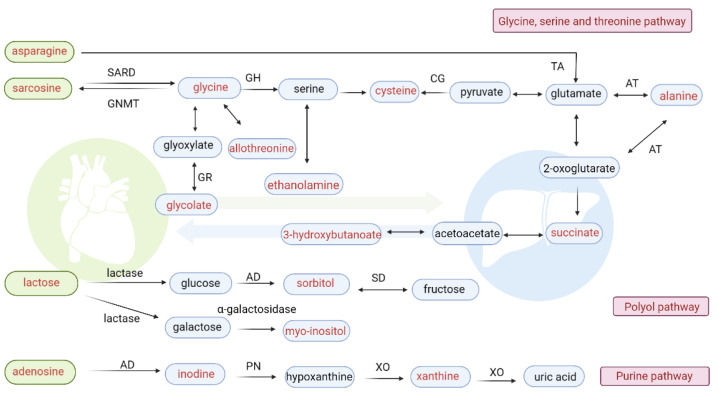
Correlation metabolic networks of main differential metabolites. The metabolites marked in red denote the main differential metabolites detected in this work. The metabolites in blue boxes were identified in hepatic tissue, whereas the metabolites in green boxes were identified in myocardial tissue. AD: adenosine deaminase, AR: aldose reductase, AT: alanine transaminase, CG: cystathionine, GH: glycine hydroxymethyl transferase, GNMT: glycine N-methyltransferase, GR: glyoxylate reductase, PN: polynucleotidase, TA: threonine aldolase, SARD: sarcosine dehydrogenase, SD: sorbitol dehydrogenase, XN: xanthine oxidase.

We also observed a decrease in ethanolamine in rats with MI. Ethanolamine is a biogenic amine found naturally in the body as part of membrane lipids and as a metabolite of the cardioprotective substances sphingosine-1-phosphate (S1P) and anandamide. Kelly et al. [35] reported that ethanolamine reduced myocardial injury caused bymyocardial ischemia-reperfusion via activation of the signal transducer and activator of transcription 3. Figure 4 shows a potential correlation between ethanolamine and maltotriose in myocardial tissue, also suggesting that ethanolamine may be involved in the cardioprotection during MI. However, no other metabolites in the sphingosine metabolism pathway were observed in this work. Thus, the effect of leonurine on sphingosine metabolism should be further confirmed by an LC-MS-based metabolomics approach. The network of relevant metabolic pathways for major differential metabolites is demonstrated in Figure 5. All the differential metabolites in this study should be further confirmed by comparison with standard substances and, where possible, verified in human source samples. Related pathways will be tested in our future work.

## 5. Conclusions

In this study, six differential metabolites were detected in myocardial tissue and twenty-five in hepatic tissue, indicating that leonurine has a greater influence on hepatic metabolism during MI. Among the differential metabolites, sarcosine was observed in both myocardial and hepatic tissue. The result of correlation analysis elucidated that some hepatic metabolites (3-hydroxybutyric acid, ethanolamine, and allothreonine) were closely related to some myocardial metabolites (maltotriose, lactose, and asparagine), suggesting crosstalk between hepatic and myocardial metabolism during MI. Leonurine showed regulation of multiple hepatic metabolic pathways, including glycine, serine and threonine, purine, fatty acids, and amino acid metabolism pathways. Among these, the glycine, serine, and threonine pathways in hepatic tissue have been identified as important cardioprotective pathways for leonurine. This study attempts to interpret the mechanism of action of leonurine against MI in terms of liver metabolism, thus laying the foundation for its future application in anti-myocardial ischemia.

## Data Availability

The data presented in this study are available in references and supplementary materials.

## Supplementary Materials

The following supporting information can be downloaded at: https://www.mdpi.com/article/10.3390/biom12101512/s1, Figure S1: A: Typical cross-sections of rat myocardium in the sham operation (S) group, MI (M) group, and leonurine administration (L) group. Areas stained red indicate normal myocardium, whereas areas not stained red and pale are infarcted. B: Effect of leonurine on myocardial infarction size in rats with MI. C and D: Effects of leonurine on plasma CK-MB activity and Tn-I concentration in rats with MI; Figure S2: Verification plots of 200 permutation tests for OPLS-DA. A: Plots for S and M groups of the myocardial (R2 = (0.0, 0.899), Q2 = (0.0, 0.265)) and hepatic tissue (R2 = (0.0, 0.877), Q2 = (0.0, 272)); B: Plots for M and L groups of the myocardial (R2 = (0.0, 0.925), Q2 = (0.0, 0.245)) and hepatic tissue (R2 = (0.0, 0.886), Q2 = (0.0, 0.198)). S: Sham-operated group; M: Myocardial ischemia model group; L: Leonurine administration group. The green circle and blue box stand for R2 and Q2, respectively; Table S1: Detailed information of thirty-one differential metabolites detected in myocardium and hepatic tissue.
