# Supplementary material for: Cardioprotective Mechanism of Leonurine against Myocardial Ischemia through a Liver–Cardiac Crosstalk Metabolomics Study
Source: Biomolecules. 2022 Oct 19;12(10):1512. doi: 10.3390/biom12101512 (PMC9599793; doi:10.3390/biom12101512)
Supplement: Supplementary file 1 [file biomolecules-12-01512-s001.zip › biomolecules-1935110-supplementary.pdf]

Supplementary Material

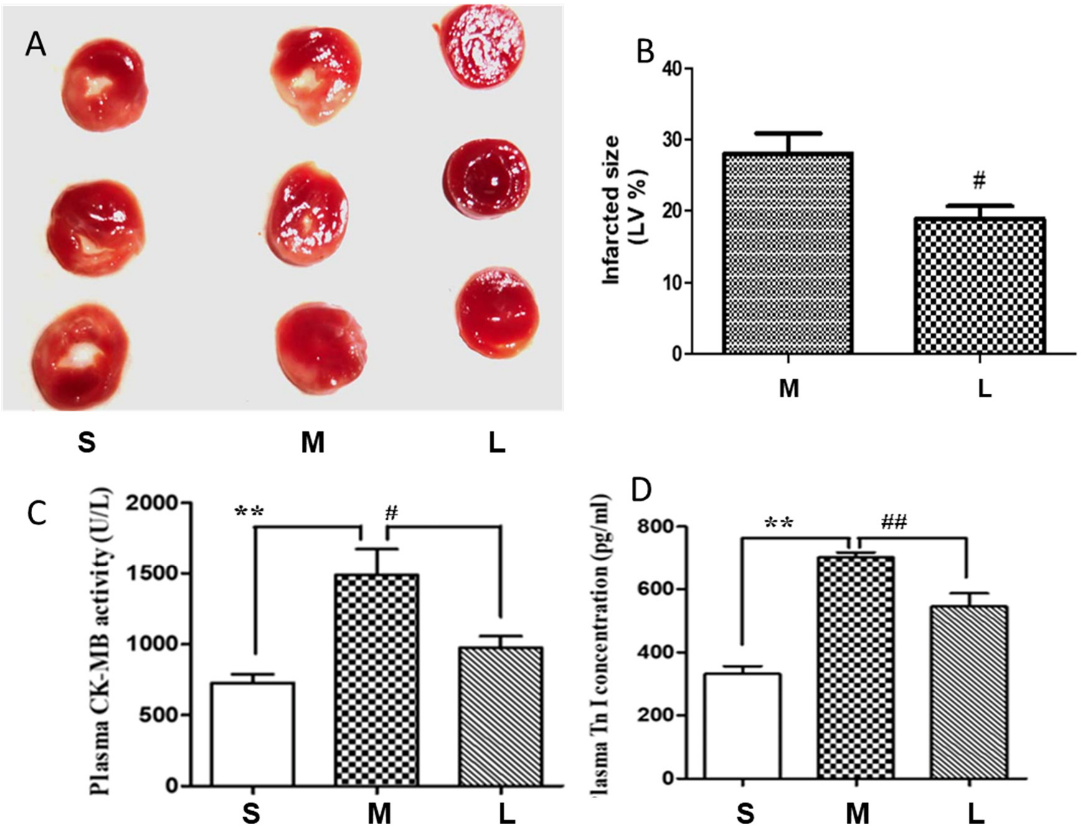

Figure S1 A: Typical cross sections of rat myocardium in the sham operation (S) group, the MI (M) group and the leonurine administration (L) group. Areas stained red indicated normal myocardium, while areas not stained red and that were pale were infarcted. B: Effect of leonurine on myocardial infarction size in rats with MI. C and D: Effects of leonurine on plasma CK-MB activity and Tn-I concentration in rats with MI. S to M: $**p < 0.01$; L to M: $\#p < 0.05$ and $\#\#p < 0.01$

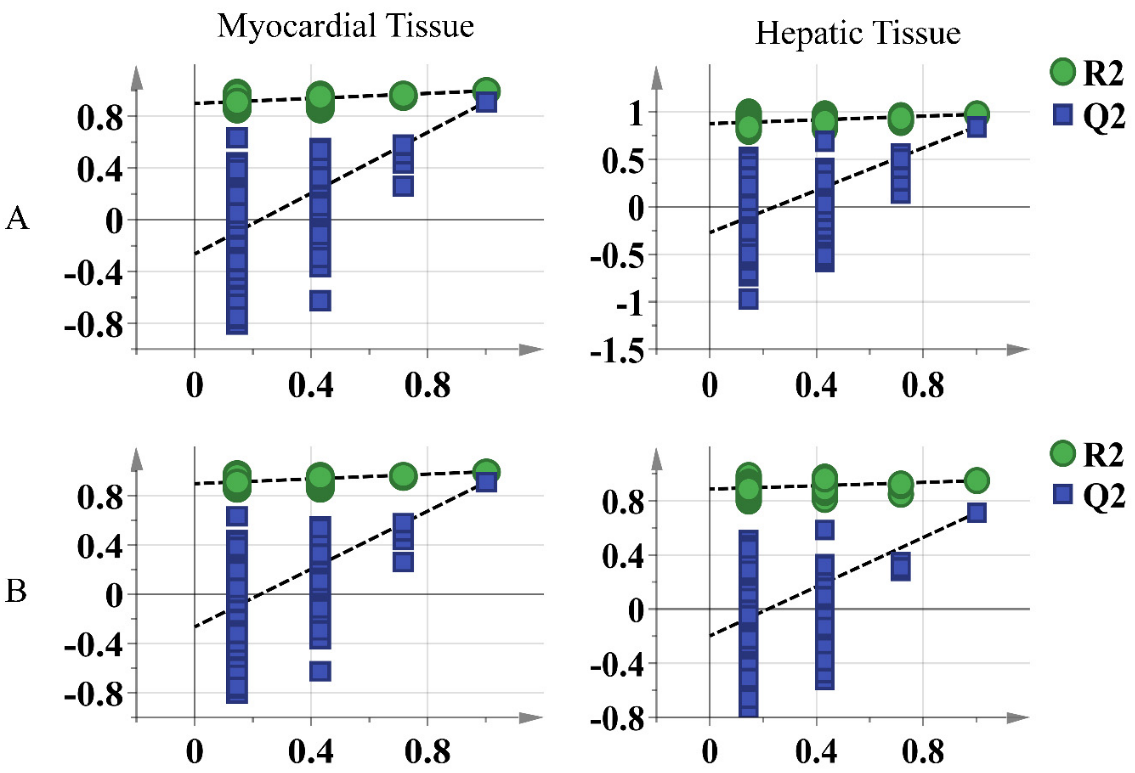

Figure S2 Verification plots of 200 permutation tests for OPLS-DA. A: Plots for S and M groups of the myocardial (R^2=(0.0, 0.899), Q^2=(0.0, −0.265)) and hepatic tissue (R^2=(0.0, 0.877), Q^2=(0.0, −.272)); B: Plots for M and L groups of the myocardial (R^2=(0.0, 0.925), Q^2=(0.0, −0.245)) and hepatic tissue (R^2=(0.0, 0.886), Q^2=(0.0, −0.198)). S: the sham-operated group; M: the myocardial ischemia model group; L: the leonurine administration group. Green circle and blue box stand for R^2 and Q^2, respectively.

Table S1 The detailed information of thirty-two differential metabolites detected in myocardium and hepatic tissue

ID	Identification	Tissue	MASS	FC (N to M)	FC (L to M)	VIP (N to M)	VIP (M to L)	Pathway
1	sarcosine	H	116	1.71*	2.18#	1.24	1.68	Glycine, serine and threonine metabolism
2	Threose	H	201	3.29**	1.98##	1.51	1.71	Energy metabolism
3	asparagine	H	146	0.61*	0.60#	1.25	1.52	Amino acids metabolism
4	maltotriose	H	204	2.22*	2.15#	1.33	1.62	Energy metabolism
5	adenosine	H	267	2.96*	1.96#	1.28	1.17	Purine metabolism
6	lactose	H	204	9.05*	9.56#	1.23	2.42	Energy metabolism
7	glycolic acid	L	147	1.92**	1.61#	1.16	0.89	Other metabolism
8	sarcosine	L	116	3.64**	2.70##	1.73	1.39	Glycine, serine and threonine metabolism
9	alanine	L	116	2.85**	2.16##	1.54	1.36	Amino acids metabolism
10	3-hydroxybutyric acid	L	147	1.91*	1.82##	1.00	1.12	Other metabolism
11	valine	L	144	2.35**	1.73##	1.38	1.13	Amino acids metabolism
12	Ethanolamine	L	174	2.73**	1.65##	1.55	1.33	sphingosine metabolism
13	proline	L	142	1.72*	1.96##	1.02	1.26	Amino acids metabolism
14	succinic acid	L	341	2.16*	3.33##	1.05	2.09	Energy metabolism
15	glycine	L	174	2.08**	3.60##	1.31	1.35	Glycine, serine and threonine metabolism
16	Allothreonine	L	219	1.56*	2.61##	0.83	1.50	Amino acids metabolism
17	cysteine	L	240	2.25*	2.23#	1.14	1.23	Glycine, serine and threonine metabolism
18	Dehydroascorbic Acid	L	264	1.77**	1.69#	1.07	1.02	Other metabolism
19	mannose	L	73	6.57**	4.46##	2.09	1.73	Energy metabolism

20	sorbitol	L	205	3.77**	2.35##	1.80	1.35	Energy metabolism
21	tyrosine	L	218	4.21**	2.09##	1.83	1.18	Amino acids metabolism
22	ascorbate	L	332	3.28**	2.23#	1.55	1.21	Other metabolism
23	lysine	L	174	4.03**	2.36##	1.79	1.35	Amino acids metabolism
24	pantothenic acid	L	103	4.05**	2.35##	1.61	1.52	Other metabolism
25	xanthine	L	353	3.79**	2.44##	1.59	1.49	Purine metabolism
26	palmitoleic acid	L	117	4.60*	2.66##	1.19	1.77	Fatty acids metabolism
27	palmitic acid	L	117	3.21**	2.17#	1.62	1.22	Fatty acids metabolism
28	myo-inositol	L	73	4.05**	2.99##	1.80	1.60	Other metabolism
29	stearic acid	L	117	2.64**	2.04#	1.37	1.13	Fatty acids metabolism
30	arachidonic acid	L	79	3.22**	2.57#	1.56	1.25	Fatty acids metabolism
31	inosine	L	73	2.93**	2.28#	1.50	1.27	Purine metabolism

S to M: **p* < 0.05 and ***p* < 0.01; L to M: #*p* < 0.05 and ##*p* < 0.01; FC: fold change. H: heart ; L: liver
